# Distribution and age of onset of psychopathological risk in a cohort of children with Down syndrome in developmental age

**DOI:** 10.1186/s13052-019-0672-4

**Published:** 2019-07-26

**Authors:** M. Marino, I. Scala, O. Scicolone, P. Strisciuglio, C. Bravaccio

**Affiliations:** Department of Medical and Translational Science, Federico II University Hospital, Via Sergio Pansini, 5, 80131 Naples, Italy

**Keywords:** Attention deficit/hyperactive disorder, Autism Spectrum disorder, Down syndrome, Oppositional defiant disorder;psychopathological risk

## Abstract

**Background:**

Aim of the study is to intercept specific characteristics and psychiatric comorbidity in Down Syndrome (DS). The study describes the distribution and the age of specific aspects of behavioral phenotype in a sample of subjects with DS.

**Methods:**

Psychopathological risk has been evaluated in a 97 DS patient cohort, aged 1 to 18 years, during regular follow-up neuropsychiatric visit and through administration of Child Behavior Checklist (CBCL); Childhood Autism Rating Scale (CARS-T) was assessed to verify the presence of autistic behaviors.

**Results:**

The results show the presence of specific psychopathological risk factors in 90% of the sample. 7% of sample presents autistic features. The risk of psychopathology is independent of the degree of intellectual disability.

**Conclusion:**

The high frequency of psychopathological risk factors indicates the need for accurate monitoring to intercept specific characteristics, such as in the case of comorbidity for autism. The search for specific psychopathological factors is a little explored aspect to date, as evidenced by the literature. Despite the studies available to date highlight the presence of psychopathological vulnerability in DS, so far there are only few reports that explore this issue systematically.

## Background

Down syndrome (DS) is a genetic condition caused by the triplication of human chromosome 21, that occurs in 1/800 live births [10]. Children and adolescents with DS have a distinctive cognitive and behavioral profile, with typical deficits in verbal communication, cognitive function, reasoning and executive verbal-related function. In comparison, non-verbal cognitive functioning is less impaired, as in the case of developing practical adaptive capabilities [13]. In almost all cases, sociability and affectivity are entirely preserved and a very cheerful and sociable temperament often characterizes people withDS [4, 8]. If compared with other children with intellectual disability (ID), children with DS are at lower risk for psychopathology [5, 10, 14]. The psychopathological profiles of DS are distinct relative to those of other subjects with ID. The most frequent psychopathological disorders in DSareAttention Deficit/Hyperactivity Disorder (ADHD), Oppositional Defiant Disorder (ODD), Autistic Spectrum Disorder (ASD), anxiety and mood disorders. In adulthood, the subjects with DS develop early aging and a high risk of the Alzheimer’s typedementia [2, 18]. During the childhood, there is a lower risk for psychopathology. During the second childhood and in youth, externalizing behaviors may be problematic, whereas a shift toward internalizing behaviors emerges with maturity [10]. Despite the studies available to date highlight the presence of psychopathological vulnerability in DS, there are only few reports that explore age-related risk factors and the distribution of specific psychopathological characteristics [3, 11, 12].

The aim of this study is to describe the distribution and the age of onset of the psychopathological characteristics of a DS patient cohort aged 1 to 18 years.

## Methods

Ninety-seven patients (46 females and 51 males, mean age: 12 years), in follow up at the Department of Medical and Translational Science of Federico II University in Naples, from February 2015 to January 2017, were evaluated for the presence of psychopathological risk factors and to defined behavioral phenotype. The Department is a regional reference center for DS and evaluation was performed during regular follow-up neuropsychiatric visit and through administration of Child Behavior Checklist (CBCL), a self-assessment questionnaire compiled by the patient’s parents for the assessment of the psychopathological risk. Childhood Autism Rating Scale (CARS-T), a rating scale used to assess the presence of autistic behaviors [1, 15], was assessed to verify the presence of autistic behaviors. These tools have been chosen for ductility, reliability and correlation with DSM-5 diagnostic criteria. Follow-up was scheduled every 6 months, during outpatients visits. They were included all patients that not have yet a diagnosed psychiatric condition and that not made psychopharmacological treatment.

Ethical approval for the study was obtained by the Ethics Committee of the University of Naples Federico II, number: 222/17.

## Results and discussion

The number of enrolled patients for each age group, mean age and sex distribution and the degree of intellectual disability are shown in Table 1.

**Table 1:** Number of patients divided by age group, age, gender and intellectual disability (ID) level

	0–2 years	2–3 years	4–5 years	6–10 years	11–14 years	15–18 years
Number	16	21	22	20	12	6
Age (Mean ± SD)	1.5 ± 0.4	2.6 ± 0.5	4.5 ± 0.5	8 ± 1.3	12.8 ± 0.9	16.5 ± 0.9
Sex (M/F)	11/6	11/10	14/8	12/8	4/8	2/4
ID level	70 ± 13	62 ± 7.9	51 ± 15	64 ± 11	50 ± 14	52 ± 16

To assess ID level, developmental quotient was used in the case of children aged less than 8 and/or with moderate intellectual disability; Intelligent quotient was used in the case of children aged > 8 and/or with mild or absent intellectual disability

**Fig. 1:**
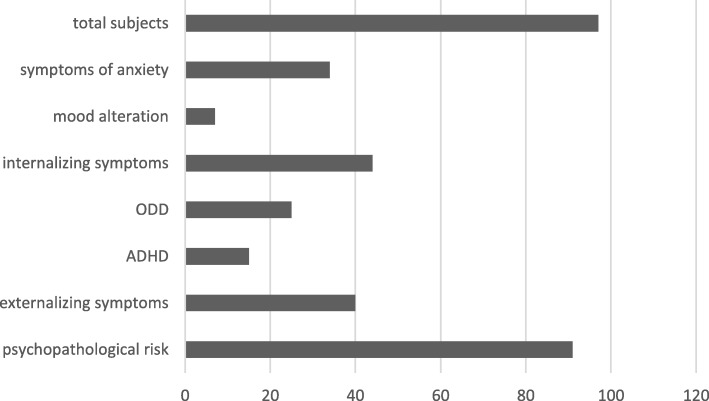
Psychopathological risk factors in the DS cohort

**Fig. 2:**
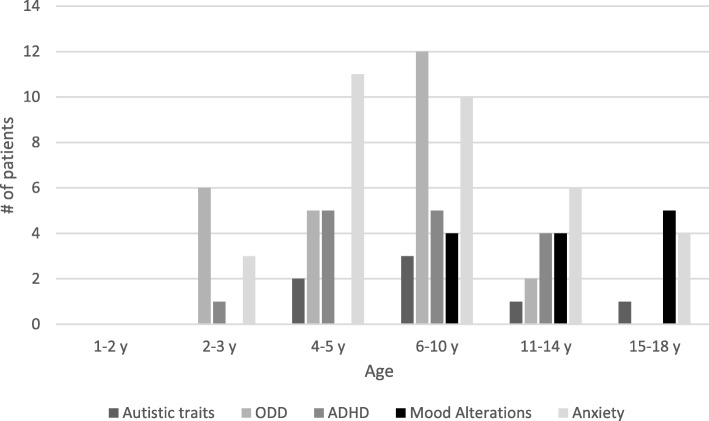
Psychopathological risk factor for age-groups

Ninety-one of 97 subjects (94%) showed psychopathological risk factors. Seven subjects (7%) had a cut-off overcoming (score > 30) for the presence of autistic behaviors (ASD) at the CARS-T rating scale, confirmed at the neuropsychiatric examination. Forty subjects (41%) had clinical scores (T score > 70) for the presence of externalization symptoms, by CBCL questionnaire, confirmed at neuropsychiatric examination. Of these, 15 (15%) showed clinical scores for the presence of ADHD symptoms, 25 (26%) for the presence of ODD symptoms. Forty-four subjects (45%) had clinical scores for the presence of internalizing symptoms by CBCL questionnaire, confirmed at neuropsychiatric examination. Of these, 9 subjects (9%) had clinical scores for the presence of mood alteration symptoms; 35 subjects (36%) had clinical scores for the presence of anxiety symptoms (Figs. 1 and 2).

In the 0–2 years age group, we found no evidence of autistic behaviors, opposite provocative disorders, attention deficit/hyperactivity disorders nor externalizing or internalizing symptoms. However, consistent with the literature, we observed sleep disorders in 25% of cases (4 out of 16) [7] and mastication dysfunction in 12.5% of children (2 out of 16) [6]. Between 2 and 3 years of age, ODD behavior and anxiety arise. Between 4 and 5 years, ODD behavior is present, hyperactivity and inattention are more relevant and anxiety has a high level of expressiveness. Between 6 and 10 years, ODD behavior and anxiety predominate, hyperactivity and inattention persist and increases the presence of mood alteration. From 11 years old, externalizing-like behavior decreases while the prevalence of anxiety and depression symptoms increases.

Compared with the mild incidence of psychopathology described in literature, the percentage of risk factors of this study is high: 94% of the sample. In [19], Visootsak et al. estimated the incidence of psychopathological characteristics in DS in a percentage between 20 and 40% of affected subjects [19]. The study by Van Gameren-Oosterom et al. on 513 adolescent patients with DS showed a percentage of behavior problems of 51% [16, 17]. The high prevalence of psychopathological risk factors in our sample suggests the need for accurate neuropsychiatric monitoring over the life span, also to identify predictors of psychopathology in the long term [11, 12, 18]. Moreover, in the studies exploring the cognitive and behavioral characteristics of subjects with DS there is much more attention to the analysis of cognitive and neuropsychological functions than those related to emotional and behavioral functioning. In [10], Grieco et al., highlighted the need of more studies to address emotional, behavioral and psychopathological aspects [10]. The data emerged from this work, therefore, suggest a high incidence of psychopathological risk, in spite of literature data that report only a moderate incidence of psychopathology. As reported in the literature, in this sample no significant psychopathological risk factors were detected in the first 2 years of life; however, during this age range, as emerged during the neuropsychiatric examination, a moderate impairment in emotional and behavioral regulation can occur and intolerance to frustration. This could be explained, in part, in relation to the global maturation process delay. These early childhood features should be explored, in our opinion, in order to evaluate their long-term impact [9]. In this sample, between 4 and 5 years of age, ODD behavior and anxiety dominate. The association, in DS children, between ODD and anxiety behavior suggests the possibility that dysfunctional behavior is reactive to an emotional dimension marked by insecurity and anxiety, poorly mentalized and regulated, also because of ID. Moreover, the difficulty to express their emotions facilitates the presence of oppositional and impulsive behaviors, also as a dysfunctional and unconscious way for controlling the environment, or for shifting the focus on their behavior rather than on the difficulties underlying them. In this age range, the greater relevance of symptoms of ADHD may be partly explained by these factors: with growth, the role of cognitive and executive functions in controlling mental and behavioral processes becomes more complex and crucial in defining a good functioning from a deficit functional level. The processes of attention, concentration and motivation mature, allowing progressively greater stability and greater control capability. A delay in maturation of these processes, as often occurred in DS, can play a key role in the development of symptoms of inattention and hyperactivity; moreover, with the growth and the schooling, environmental demands increase, so the impairment is more evident. Between 6 and 10 years the presence of mood alteration increases: this is in part correlated to the psychological growth, which, especially in DS subjects with better cognitive competence, involves a greater awareness of their condition and of the related difficulties. Moreover, at this age, the perception of themselves and other individual characteristics begins to develop; this can, in general, make the peer group less inclusive toward a child with disability, making integration and social contacts more difficult. These elements can undoubtedly have an impact on mood and on behavior, leading at the increase of depressive symptoms. In this sample, 7 subjects (7%) have a cut-off overcoming (score > 30) for the presence of autistic behaviors (ASD) at the CARS-T rating scale, confirmed at the neuropsychiatric examination. DS subjects with autism symptoms have peculiar features: increased stereotypes, higher level of anxiety, and increased adaptive impairment, if compared to typical autism. The presence of these specific characteristics in the subjects of this sample, consistent with the hypothesis of a specific autistic phenotype in Down’s syndrome, is an important preliminary data to be further analyzed.

This element is, in our view, very important, because it underlines the importance of using specific screening tools to identify specific psychopathological features, otherwise unidentified or merely attributed to secondary aspects of mental retardation. The diagnosis of specific traits of autism in DS is relevant from a clinical point of view, but very complex. Dressler et al., in a 2011 study on adaptive behavior of subjects with DS and autism, showed how autism in DS is often underestimated in the clinical context, because it is often assimilated to characteristics related to ID [2]. As highlighted in this paper, the need to screen specifically comorbidity between Down syndrome and Autism is relevant to a clinical and therapeutic point of view.

In our sample, the degree of intellectual disability (moderate vs mild intellectual disability) was not associated with increased risk of behavioral disorders (Chi2 1.2; p 0.2). No patient with severe intellectual disability was present in our cohoort.

Hence, in this study, the risk of psychopathology is independent of the degree of intellectual disability.

## Conclusions

Data emerged from this study suggest a high incidence of psychopathological risk across the evolutionary age in DS. The results of this report highlight the necessity for accurate neuropsychiatric monitoring of subjects with DS during development, based on the evidence of a high percentage of psychopathological risk and on the possibility to identify specific characteristics related to psychopathological expressiveness. Our results also highlight the importance of using in clinical practice specific screening tools, to identify psychopathological risk factors, pointing up some qualitative and specific features, otherwise unidentified or merely attributed to secondary aspects of mental delay. Additional studies on larger DS cohort using of a more extensive psychodiagnostic protocol and of a data related to I.Q. will be needed to thoroughly explore the risk factors highlighted so far.

## Data Availability

The authors declare that the data needed to support and explain the findings of this study are available and deductible within the article and its tables.

## Abbreviation

ADHD: Attention Deficit/Hyperactive Disorder
ASD: Autism Spectrum Disorder
CARS – T: Childhood Autism Rating Scale
CBCL: Child Behavior Checklist
DS: Down syndrome
ID: Intellectual disability
IQ: Intellectual quotient
ODD: Oppositional Defiant Disorder
